# Five years’ retrospective analysis of childhood ocular morbidities: A priority setting guidelines for pediatric eye clinic

**DOI:** 10.12669/pjms.38.6.5441

**Published:** 2022

**Authors:** Sadia Bukhari, Shua Azam, Shahid Ahsan, Tauseef Mahmood, Muhammad Saleh Memon, Uzma Haseeb, Muhammad Arslan

**Affiliations:** 1 Dr. Sadia Bukhari, (MS Ophth). Department of Ophthalmology, Al Ibrahim Eye Hospital, Isra Postgraduate Institute of Ophthalmology, Karachi, Pakistan; 2 Ms. Shua Azam, M.Phil. (Optometry). Isra School of Optometry, Al Ibrahim Eye Hospital, Isra Postgraduate Institute of Ophthalmology, Karachi, Pakistan; 3 Dr. Shahid Ahsan, M.Phil. (Bio), M.Phil (NCD), PhD Fellow. Department of Biochemistry, Jinnah Medical & Dental College, Karachi, Pakistan; 4 Mr. Tauseef Mahmood, M.Sc. (Statistics). Department of Research, Al Ibrahim Eye Hospital, Isra Postgraduate Institute of Ophthalmology, Karachi, Pakistan; 5 Dr. Muhammad Saleh Memon, FRCS (Eden). Department of Research, Al Ibrahim Eye Hospital, Isra Postgraduate Institute of Ophthalmology, Karachi, Pakistan; 6 Dr. Uzma Haseeb, (FCPS). Department of Ophthalmology, Al Ibrahim Eye Hospital, Isra Postgraduate Institute of Ophthalmology, Karachi, Pakistan; 7 Mr. Muhammad Arslan, (MCSW). Department of Research & Excellence, Al-Tibri Medical College, Karachi, Pakistan

**Keywords:** Conjunctivitis, Eye disease, Pediatric ophthalmology, Primary eye care, Refractive error

## Abstract

**Objectives:**

To observe patterns of Pediatric eye diseases over five years 2015-19, to improve management of ophthalmic pediatric units in the developing countries.

**Methods:**

It was an observational, cross-sectional study carried out in a tertiary eye care Hospital, Karachi. Records of the children under 16 years of age from 2015 to 2019 were retrieved. Inclusion criteria included complete records with age, gender of the children, symptoms, examination, investigation if necessary, and diagnosis. All incomplete records were excluded.

**Results:**

A total of 35348 records with 55.17% boys and 44.82% girls were analyzed. Similar gender difference was reflected in disease frequency. Seven percent of the children did not have detectable ocular pathology. Conjunctivitis, refractive errors and squint were the three most common ocular morbidities observed in decreasing order of frequency as 32.67%, 20.08% and 14.7% respectively. Cataract was present in 4.51%, Corneal disease in 4.11%, Retinal pathology in 1.04%, Glaucoma in 0.49% cases; but Retinoblastoma was present in 55 cases and ROP in 4 cases only. Almost 60% of the children had simple ocular problems like conjunctivitis, refractive error and absence of any pathology.

**Conclusion:**

Majority of the children attending pediatric ophthalmology had simple problems manageable at primary health facility level. Strengthening of the primary health care facility will reduce considerable burden of pediatric unit at the tertiary level. Optometrists and orthoptists are important members of the team for refraction and squint management.

## INTRODUCTION

Ocular morbidity describes spectrum of eye diseases experienced by a population that are either significant to the individual or to eye professionals.^1^ Eye problems in children are one of the important cause of medical consultation and If not attended may lead to severe visual impairment and blindness.^2^ Visual impairment in young children delays motor, language, emotional, social and cognitive development, with lifelong consequences. School going children with visual impairment can also experience lower levels of educational achievement. Consequently, it impacts quality of life in adulthood.^3^ Especially in low income countries with less resources and deficient education they get less employment opportunities. They might also face difficulties in social interactions which can lead to social loneliness, anxiety and depression.^3^

Globally 19 million children have visual impairment with 1.26 million blind bilaterally and, an estimated 70 million blind years are caused by childhood blindness.^4^ Data for visually impaired and blind children for Pakistan is not available. The available evidence suggests that the prevalence varies from 0.3/1000 children in economically developed countries to over 1.0/1000 children in underdeveloped societies.^5^ Population of children under 15 years is 90 million^6^ (43.4% of country’s population). One can project number of visually impaired children at 1.0/1000 children as 0.09 million or 90000 children. Almost half of all blindness in children particularly those in the poor countries is due to avoidable causes that are amenable to cost effective interventions.^7^ The goal of VISION 2020 recommends one ophthalmologist trained in pediatric eye diseases for every 10 million people by 2020.^8^ There are enough practicing pediatric ophthalmologist but very few properly trained and experienced pediatric ophthalmologist. All the tertiary centers are trying to develop pediatric ophthalmology units and need equipment and human resource. Nationally there is no guide line for such development. This study intends to develop these guide lines.

In the past few years’ childhood ocular morbidity is dominated by allergic conjunctivitis and refractive errors.^9^ Studies from Pakistan also reported similar pattern of ocular problems.^10^,^11^ Eye trauma in children is common cause of unilateral severe visual impairment and cosmetic disfigurement resulting in psychological impact on personality and behavior.^12^

This study will give us baseline data of pediatric ocular morbidities in children attending tertiary eye care centre, Karachi. This baseline data will provide clinical based evidence for the relevant authorities to formulate a policy to reduce the burden on tertiary eye care hospitals and provide chances to the pediatric ophthalmologist to concentrate on training, teaching and research in addition to the clinical work. This study will also draw attention of institutional heads to identify the areas of efficient allocation, investment and prioritization of financial as well as human resources.

## METHODS

It was an observational, cross-sectional study with retrospective data collection retrieved from Hospital Information Management System (HIMS) of Al Ibrahim Eye Hospital, Karachi from Jan 2015 to Dec 2019. A prior Ethical Approval was taken from Institute Research Ethical Committee. Study protocol number was A-00094. Non-probability purposive sampling technique was used for sample collection from software. Inclusion criteria was clinical records of all children aged up to 15 years attending outpatient department of Pediatric Ophthalmology unit were retrieved irrespective of age, gender and ethnicity. Missing records or unclear diagnosis with incomplete data were excluded from the study.

### Data Collection Procedure

All these patients underwent detailed examination including History taking, checking visual acuity with the help of recommended tools including Central, steady and maintained (CSM), Lea Gratings, Cardiff visual acuity cards and Snellen’s chart. Cycloplegic refraction was done when needed. Complete Ophthalmic examination was carried out with the help of direct ophthalmoscope, slit lamp (Hand held slit lamp was used in younger children), +90 D lens and indirect ophthalmoscope were used for examination of fundus. B-scan ultrasound performed in cases where fundus view was not clear. Examination under sedation or general anesthesia was carried out where needed. After detailed clinical examination diagnosis was made and recorded.

### Statistical Analysis

Data was retrieved from HIMS software and exported to SPSS version 23.0 for data analysis. Mean and Standard deviation was calculated for continuous variables. Frequency and percentages were reported for categorical variables like gender, age groups and diagnosis. Bar chart was made to present age groups. Cross tabulation was done between diagnosis with gender and age groups using Chi-square test. P-value ≤ 0.05 considered to be statistically significant.

## RESULTS

Complete records of 35348 children visiting pediatric clinic of Al-Ibrahim Eye Hospital with different ocular problems were retrieved. Mean age of the patient was 7.58 ± 4.42 years. Gender distribution was observed as boys 19503 (55.17%) and girls 15845 (44.82%). Boy to girl ratio was 1.2:1.

**Fig.1:**
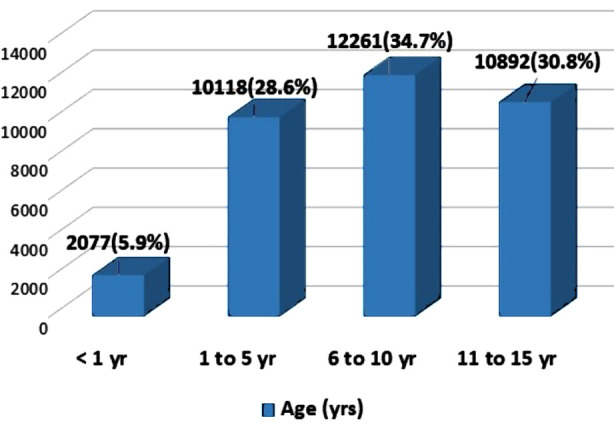
Age Distribution.

Among four age groups, 2077 (5.9%) patients were of age less than one year, 10118 (28.6%) were in between one to five years. Frequency of the children in the third age group (6-10 years) was 12261 (34.6%) and 10892 (30.8%) patients were in between 11 to 15 years (Fig.1).

Conjunctivitis with 11550 (32.67%) children was commonest disease. Amongst conjunctivitis, allergic was commonest (37.6%), bacterial was found in 37.5%, vernal conjunctivitis 14.42%, viral 5.67% and 4.77% were labeled nonspecific. Second commonest cause was Refractive error which was found in 7100 (20.08%) children. Hypermetropia found was 45.75%, myopia 33%, astigmatism 18%. Children found to be amblyopic were 3.38% (Table-I). The third common cause was squint 5198 (14.70%). Children who did not have any visible disease were 2476 (7%). Cataract was present in 1597 (4.51%), Cornea affected in 1461 (4.11%), Retinal disease in 370 (1.04%). Glaucoma was present in 176 (0.49%) cases. Boys’ dominance was easily seen in conjunctivitis as 6778 (19.17%) against 4772 (13.50%) girls. Similarly, more boys 3625 (10.25%) compared to girls 3475 (9.83%) were diagnosed with different types of refractive error. Likewise, Squint cases had a slightly upper share 2677 (7.57%) in boys as compare to girls 2521 (7.13%) (Table-II).

**Table I:** Proportion of Conjunctivitis and Refractive Error.

Conjunctivitis	Count	%
Allergic Conjunctivitis	4344	37.61
Bacterial Conjunctivitis	4335	37.53
Vernal Keratoconjunctivitis (VKC)	1665	14.42
Viral Conjunctivitis	655	5.67
Nonspecific conjunctivitis	551	4.77
Total	11550	100

*Refractive Error*	*Count*	*%*

Hypermetropia	3245	45.7
Myopia	2338	32.92
Astigmatism	1469	17.99
Amblyopia	240	3.38
Total	7100	100

**Table II:** Gender wise diagnosis.

Diagnosis	Gender			P-value

	Boys	Girls	Total	
Conjunctivitis	6778 (19.17%)	4772 (13.50%)	11550 (32.67%)	0.001
Refractive Error	3625 (10.25%)	3475 (9.83%)	7100 (20.08%)	
Squint	2677 (7.57%)	2521 (7.13%)	5198 (14.70%)	
Normal Quite Eye	1251 (3.53%)	1225 (3.46%)	2476 (7%)	
Eye Lid Pathology	1052 (2.97%)	1112 (3.14%)	2164 (6.12%)	
Lacrimal Systematic Disease	948 (2.68%)	889 (2.51%)	1837 (5.20%)	
Cataract	1039 (2.93%)	558 (1.57%)	1597 (4.51%)	
Corneal diseases	913 (2.58%)	548 (1.55%)	1461 (4.13%)	
Trauma	323 (0.91%)	170 (0.48%)	493 (1.39%)	
Retinal diseases	245 (0.69%)	125 (0.35%)	370 (1.04%)	
Glaucoma	100 (0.28%)	76 (0.21%)	176 (0.49%)	
Orbital diseases	89 (0.25%)	80 (0.22%)	169 (0.47%)	
Dry Eye	90 (0.25%)	64 (0.18%)	154 (0.43%)	
Optic Nerve diseases	73 (0.20%)	51 (0.15%)	124 (0.35%)	
Developmental Anamolies	50 (0.14%)	27 (0.07%)	77 (0.21%)	
Pthysis Bulbi	40 (0.11%)	14 (0.04%)	54 (0.15%)	
Endophthalmitis	29 (0.08%)	19 (0.05%)	48 (0.13%)	
Uveitis	20 (0.06%)	16 (0.04%)	36 (0.10%)	
Down Syndrome	11 (0.03%)	8 (0.02%)	19 (0.05%)	
Other Diagnosis	150 (0.43%)	95 (0.27%)	245 (0.69%)	
Total	19503 (55.17%)	15845 (44.82%)	35348 (100%)	

Diagnosis on the basis of age groups are presented in Table-III. Most of the cases of conjunctivitis 4150 (11.74%) belong to age group 6 to 10 years. While refractive error 3640 (10.29%) mostly found in between 11 to 15 years of age. Similarly, most of the squint cases 1999 (5.65%) found in early age of one to five years. Cataract 566 (1.60%), Cornea 562 (1.58%) and Retina 142 (0.40%) related cases were mostly found in between 6 to 10 years of age.

**Table III:** Age wise diagnosis.

Diagnosis	Age Groups				Total	P-value

	less than 1 yr.	1 to 5 yr.	6 to 10 yr.	11 to 15 yr.		
Conjunctivitis	635 (1.79%)	3721 (10.52%)	4150 (11.74%)	3044 (8.61%)	11550 (32.67%)	0.001
Refractive Error	40 (0.11%)	757 (2.14%)	2663 (7.53%)	3640 (10.29%)	7100 (20.08%)	
Squint	144 (0.40%)	1999 (5.65%)	1848 (5.22%)	1207 (3.41%)	5198 (14.70%)	
Normal Quite Eye	126 (0.36%)	603 (1.70%)	867 (2.45%)	880 (2.49%)	2476 (7%)	
Eye Lid Pathology	54 (0.15%)	585 (1.65%)	787 (2.22%)	738 (2.08%)	2164 (6.12%)	
Lacrimal Systematic Disease	807 (2.28%)	847 (2.39%)	116 (0.32%)	67 (0.19%)	1837 (5.20%)	
Cataract	111 (0.31%)	532 (1.50%)	566 (1.60%)	388 (1.10%)	1597 (4.51%)	
Corneal diseases	93 (0.26%)	440 (1.25%)	562 (1.58%)	366 (1.03%)	1461 (4.13%)	
Trauma	1 (0.003%)	160 (0.45%)	205 (0.58%)	127 (0.36%)	493 (1.39%)	
Retinal diseases	10 (0.03%)	103 (0.29%)	142 (0.40%)	115 (0.32%)	370 (1.04%)	
Glaucoma	25 (0.07%)	64 (0.18%)	43 (0.12%)	44 (0.12%)	176 (0.49%)	
Orbital diseases	11 (0.03%)	84 (0.24%)	42 (0.12%)	32 (0.09%)	169 (0.47%)	
Dry Eye	1 (0.003%)	34 (0.10%)	57 (0.16%)	62 (0.17%)	154 (0.43%)	
Optic Nerve diseases	7 (0.02%)	36 (0.10%	44 (0.12%)	37 (0.10%)	124 (0.35%)	
Developmental Anamolies	2 (0.005%)	29 (0.08%)	23 (0.06%)	23 (0.06%)	77 (0.21%)	
Pthysis Bulbi	0 (0%)	10 (0.02%)	26 (0.07%)	18 (0.05%)	54 (0.15%)	
Endophthalmitis	1 (0.003%)	18 (0.05%)	16 (0.04%)	13 (0.03%)	48 (0.13%)	
Uveitis	1 (0.003%)	6 (0.01%)	16 (0.05%)	13 (0.04%)	36 (0.10%)	
Down Syndrome	1 (0.003%)	10 (0.03%)	2 (0.005%)	6 (0.01%)	19 (0.05%)	
Other Diagnosis	7 (0.02%)	80 (0.23%)	86 (0.24%)	72 (0.20%)	245 (0.69%)	
Total	2077 (5.87%)	10118 (28.62%)	12261 (34.68%)	10892 (30.81%)	35348 (100%)	

## DISCUSSION

This study validates findings of earlier studies regarding childhood ocular morbidity dominated by allergic conjunctivitis and refractive errors.^10^,^11^ The data shows that over a period of 5 years, conjunctivitis was present in 32.67% children. Of this 37.6% were allergic and 14.4% were VKC. Study from Sind Province reported 34.1% conjunctivitis cases in children attending 10 BHUs.^13^ In a study from Punjab Province reported 22.1% VKC and 34% refractive errors.^11^ Study from Karachi reported 24.8% VKC and 15.2% refractive errors.^12^ Another tertiary center in Bahawalpur^13^ reported 32.2% conjunctivitis and 21.9% refractive errors. A study from Ethiopia showed 30.5% infections of conjunctiva and lid, 21.9% refractive errors and VKCs 28% and allergic conditions. There seems consensus on the findings that conjunctivitis is commonest eye disease in children in the developing countries.

Second common cause of ocular morbidity in children is refractive errors. Present study shows Refractive error in 20.08% children. Other national studies also present refractive errors as common morbidity in children with variable frequency from 33%^11^, 14.8%^12^ and 32.1%.^13^ Present study differs from majority of studies where myopia is more common. The population-based prevalence of myopia, hyperopia (≥ +2.00 D) and astigmatism in India was 5.3%, 4% and 5.4%, respectively.^14^ Study from Pakistan showed myopia as 52%, astigmatism as 38% and Hypermetropia as 10%.^15^ Our study is supported by Slaveylokov K et al.^16^ where hypermetropia was seen in 78.85%. High number of hypermetropic patients in our study is probably because of significant number of squint patients attending the clinic.

All these studies show that 60% (conjunctivitis 33%, normal eyes 7% and refractive errors 20%) children could have been treated at Basic Health Unit (BHUs) and Rural Health Centres (RHCs). Strengthening BHUs and RHCs can not only lessen the burden of pediatric eye care centers by 60% but will make the treatment of children more accessible and cost effective. Strengthening will include education of the professionals of primary eye care facility specially regarding management of allergic conjunctivitis and Vernal Keratoconjunctivitis (VKC). Patients with VKC and allergic conjunctivitis should always be told the hidden effect of steroid which are frequently used in the treatment of these two diseases. Steroid induced glaucoma^17^ and cataract^18^ are known complications of long term use of steroids leading to blindness. Apparently simple diseases become important due to the complications of treatment. Constant vigilance and education of the professionals as well patients are required. Another important emerging complication of VKC is Keratoconus.^19^,^20^ It is to be reminded to the primary health facilitators that if allergic conjunctivitis and VKC does not respond to treatment in few weeks, they are to be referred to tertiary level facility.

Remaining 40% cases need to be referred to a Pediatric unit. They can be conveniently grouped on the basis of expertise of the surgeon needed. First Group will consist of Squint (14.8%), pediatric cataract (4.57%) and ocular trauma (1.39%) which can be managed by general pediatric ophthalmologist. Second Group will include corneal diseases (4.18%), retinal problems (1.0%), congenital/developmental glaucoma (0.5%), Lacrimal (6.12%), lids (5.2%), orbit (0.47%) and advanced cases of ocular trauma which can be managed by pediatric ophthalmologist trained in particular sub-specialty.

In the first group Squint was reported as commonest pediatric problem in this study (14.8%), mostly (10.87%) in the age group 1-10 years. No gender difference was found (7.5% boys and 7.13% girls). Other studies reported almost same or near frequency, 12.4% by Farrukh S et.al from Karachi^11^, 13.5% by Sethi et al. from North West Frontier Province of Pakistan.^21^ Assessment of the squint is most important aspect of the management of squint and is best carried by orthoptist or trained optometrist. Tertiary center should have an orthoptist to manage squints.

Cataract is the most important cause of treatable blindness in childhood.^22^ National studies have reported 24.3%^9^ (BHUs, sample 1000 children) 6%^13^ (tertiary hospital with 1000 sample) and 23.1%^14^ (tertiary hospital and 1000 sample). In present study Cataract was found in 4.57% (N;1598) of the children attending a pediatric unit of Karachi during five years. The low number of pediatric cataract as compared to National statistics is probably due to missing records. Management of cataract in children has undergone tremendous change after advances in the technology like anterior vitrectomy^23^ and IOL as primary procedure^24^ or scleral fixations^25^ as secondary procedure.

Among the second group, most important problem to be addressed is the ocular trauma. There are many local studies on this subject.^16^,^26^ Ocular trauma is a leading cause of visual morbidity in children. This is preventable to certain extent by better care and supervision. Besides, direct damage to the ocular structures resulting in loss of vision, poor visual outcome may also due to dense amblyopia caused by prolonged period of visual deprivation.

Important retinal problems are retinoblastoma and retinopathy of prematurity (ROP). There were only 55 recorded retinoblastomas and four ROPs. One can only presume that these diseases may have been dropped as incomplete records. None of the national studies cited above have reported Retinoblastoma and ROPs.

### Recommendations

Strengthening BHUs and RHCs can not only decrease the burden of pediatric eye care centers by 60%; but will make the treatment of children more accessible and cost-effective. Constant vigilance and education of professionals including pharmacy personals as well patients are required in prescription and usage of steroids. It is to be reminded to the primary health facilitators that if allergic conjunctivitis and VKC does not respond to treatment in couple of months, they are to be referred to tertiary level facility. Optometrist and orthoptist should be included in the pediatric ophthalmic team. Tertiary care units of Pediatric ophthalmology should include specially trained ophthalmologists (including pediatric oncologist) who can deal with cases of pediatric cornea, glaucoma, retina, and pediatric ocular malignancies. Pediatric ophthalmology units should also include facilities of low vision clinics to deal with children who are permanently blind including their rehabilitation academic and career counselling. Pediatric ophthalmology units should primarily be equipped by Phaco and vitrectomy machines. Secondly digital fundus camera and indirect ophthalmoscope with laser should be acquired.

### Limitations

Although we have representation from different ethnic backgrounds but most of the patients were from Sindh and Baluchistan. It’s retrospective data from one of the large tertiary care hospitals in Pakistan but it is a single-center data and future publications are needed incorporating data from multiple tertiary care centers. Also, our center is a pure eye center thus the systemic diseases might have less frequency documented than the actual numbers.

## CONCLUSION

Majority of the children attending pediatric department are simple diseases to be treated at primary eye care level if strengthened by optometrist and primary level ophthalmologist. For prevention and early diagnosis of blindness due to ROP, retinal digital imaging should be made available at pediatric ophthalmology units of tertiary care eye hospitals.

### Authors’ Contribution:

**Sadia Bukhari:** Concept of study, methodology writing and critical review.

**Shua Azam:** Introduction and literature search.

**Shahid Ahsan:** Final review.

**Tauseef Mahmood:** Statistical analysis and result write-up.

**Muhammad Saleh Memon:** Discussion writing. He is also responsible for the integrity and accuracy of the study.

**Uzma Haseeb:** Review from a clinical point of view as an ophthalmologist.

**Muhammad Arslan:** Editing of the manuscript.
